# The Use of Mixed Methods in Research on Resilience Post Sexual Assault: An Integrative Review

**DOI:** 10.3390/nursrep15070237

**Published:** 2025-06-27

**Authors:** Louisette Abikou, Tausi Haruna

**Affiliations:** 1 College of Nursing, University of Cincinnati, 3110 Vine Street, Cincinnati, OH 45221, USA; harunats@mail.uc.edu; 2 Fundamentals of Nursing and Basic Sciences, Kairuki University, Dar es Salaam P.O. Box 65300, Tanzania

**Keywords:** resilience, coping, healing, sexual assault, rape, mixed methods, multi-methods

## Abstract

**Background:** Sexual assault affects millions globally each year, yet research on survivors’ resilience remains limited and methodologically inconsistent. Resilience after sexual assault is a multidimensional and deeply personal process shaped by psychological, social, and systemic influences. Understanding this complexity requires research methodologies that can capture both measurable patterns and lived experiences. **Objectives:** This integrative review explores how mixed-methods research (MMR) has been used to study resilience and healing following sexual assault. **Methods:** Using the framework by Whittemore and Knafl, four databases (PubMed, CINAHL, PsychInfo, and EMBASE) were searched for peer-reviewed primary research articles published between 2014 and 2024. Studies were screened and appraised independently by two reviewers using predefined inclusion and exclusion criteria and a modified Johns Hopkins Nursing Evidence-Based Practice (JHNEBP) tool. **Results:** Six studies met the inclusion criteria, revealing substantial variation in how mixed-methods approaches were applied. Half of the studies cited a “completeness” rationale for integrating qualitative and quantitative strands, while others emphasized enhancement, triangulation, or discovery of new insights. Integration strategies included connecting datasets sequentially, merging findings at the interpretation stage, or building new frameworks from combined results. However, only one study used a joint display to visually represent integration, highlighting an ongoing underutilization of visual synthesis tools in trauma research. Measures of resilience and associated outcomes also varied widely across studies, underscoring the complex and multidimensional nature of resilience following sexual assault. **Conclusions:** This review suggests that MMR can offer a deeper, more nuanced understanding of resilience among sexual assault survivors and calls attention to the need for intentional integration strategies to maximize insight.

## 1. Introduction

Sexual assault, as defined by the World Health Organization [1], refers to any sexual act, attempt to obtain a sexual act, or other act directed against a person’s sexuality using coercion, regardless of the relationship to the victim. It can include childhood sexual abuse, intimate partner violence, and institutional abuse, among others. Sexual assault often leaves deep and lasting emotional, physical, and psychological scars on survivors [1]. Yet, in the wake of such trauma, many survivors demonstrate resilience, which, for the purpose of this review, is defined as the ability to adapt, recover, and navigate healing after adversity [2]. This resilience is not simply a personal trait but is shaped by individual experiences, community support, and broader social systems [3]. Understanding how survivors build and sustain resilience is essential for developing support systems and interventions that are truly trauma-informed. For mental health nurses, understanding resilience after sexual assault is vital. Survivors often turn to mental health services for immediate and long-term support, making it essential for nurses to recognize the multidimensional processes of healing and adaptation. Mental health nursing practice stands to benefit from research that captures both psychological symptoms and the lived emotional realities of survivors, enabling more empathetic and evidence-based care.

However, studying resilience is inherently complex. Quantitative methods allow researchers to measure symptoms, trends, and predictors, while qualitative methods reveal the depth of survivors’ lived experiences, which are often rich, emotional, and nonlinear. Mixed-methods research (MMR) provides a bridge between these two worlds, combining numerical data with narrative accounts to offer a fuller understanding of the healing process [4,5]. In the context of sexual assault and trauma recovery, this methodological approach is particularly valuable, as it allows researchers to simultaneously explore measurable outcomes (such as PTSD symptoms or social support levels) and the nuanced meaning-making processes that shape survivor resilience. While several reviews have explored resilience itself (e.g., [6,7]), none to our knowledge have examined how MMR has been applied to study resilience specifically after sexual assault. This distinction matters. Mixed-methods studies have the potential to capture both the quantifiable effects of trauma and the complex emotional and relational dynamics of healing, yet without methodological clarity, this potential may go unrealized. A synthesis focused on how MMR is being used in this area can help clarify the various approaches to data collection, integration, and rationale-setting, and how those choices affect research quality and insight. This review therefore fills a critical gap in the literature by evaluating the methodological strategies of existing MMR studies on resilience after sexual assault, with the goal of guiding trauma researchers toward more intentional, rigorous, and context-sensitive mixed-methods design.

This integrative review focuses specifically on how mixed methods have been used to study resilience after sexual assault. It examines not just the types of data collected, but also the rationales for using MMR and how qualitative and quantitative findings are integrated. Integration refers to the process by which researchers combine insights from different data strands during the design, analysis, or interpretation stages of a study [8,9]. Integration can take many forms, including *connecting* (where one strand informs the sampling or questions of the other), *merging* (analyzing both datasets together), and *building* (using results from one strand to inform the development of the other). Another increasingly recommended technique involves the use of *joint displays*, which visually align qualitative and quantitative results to enhance interpretation. Each integration approach offers distinct strengths: connecting enables iterative exploration, merging supports comprehensive analysis, and building facilitates hypothesis generation. However, they also come with limitations. For instance, merging can obscure nuanced differences if not done carefully and connecting may result in imbalanced weighting between strands. Without clear and intentional integration, studies risk producing fragmented findings that fail to capitalize on the complementarity of mixed methods [8,9]. Proper integration is therefore a critical quality marker in MMR, particularly in trauma research, where both measurable and narrative insights are essential for holistic understanding.

Equally important is the rationale behind choosing a mixed-methods approach. A well-articulated rationale serves as the conceptual foundation that links research questions to methodological design. Common rationales include *completeness* (capturing a fuller picture by combining data types), *triangulation* (cross-verifying findings for credibility), *enhancement* (using one strand to deepen understanding of the other), and *discovery or explanation* (generating or clarifying unexpected insights) [10]. In the context of sexual assault and resilience research, these rationales are particularly meaningful. The ability to justify why both numerical and narrative data are needed demonstrates methodological intentionality and enhances transparency for readers and reviewers. When rationale statements are vague or absent, it becomes difficult to evaluate or justify the study design, ultimately weakening the strength of the findings. This review, therefore, places equal emphasis on the clarity of rationales and the strategies used for integration, recognizing both as cornerstones of high-quality MMR in trauma contexts.

Ultimately, the review aims to offer practical insight for trauma researchers considering mixed-methods approaches in their work. It focuses on methodological execution and is deeply grounded in the specific context of sexual assault and resilience, where both rigor and sensitivity are required.

Research Questions:

1. What rationales do researchers provide for using mixed methods in studying resilience after sexual assault?
2. What qualitative and quantitative measures or scales are employed to explore resilience in these studies?
3. How and where does integration occur in these mixed-methods studies?

## 2. Methods

### 2.1. Design

This review employed the integrative review methodology proposed by Whittemore and Knafl [11], which enables the inclusion of diverse research designs. Their five-step approach, which includes problem identification, literature search, quality appraisal, data analysis, and synthesis, was used to ensure systematic rigor while remaining flexible enough to capture methodological diversity.

### 2.2. Search Strategy

Four databases (PubMed, CINAHL, PsychInfo, and EMBASE) were searched using combinations of the following keywords: *resilience*, *coping*, *healing*, *sexual assault*, *rape*, *mixed methods*, and *multi-methods*. The search was conducted between the 20th and 24th of February, 2024. A sample search string used in PubMed was: (“resilience” OR “coping” OR “healing”) AND (“sexual assault” OR “rape”) AND (“mixed methods” OR “multi methods”). The inclusion of “multi-methods” helped capture studies that may have used different terminology. The 2014–2024 timeframe was selected to reflect the most recent decade of research following increased emphasis on trauma-informed care, the growing application of mixed methods in health and social sciences [4], and major cultural shifts such as the #MeToo movement, which reshaped the discourse around sexual violence and survivor narratives [12].

**Figure 1:**
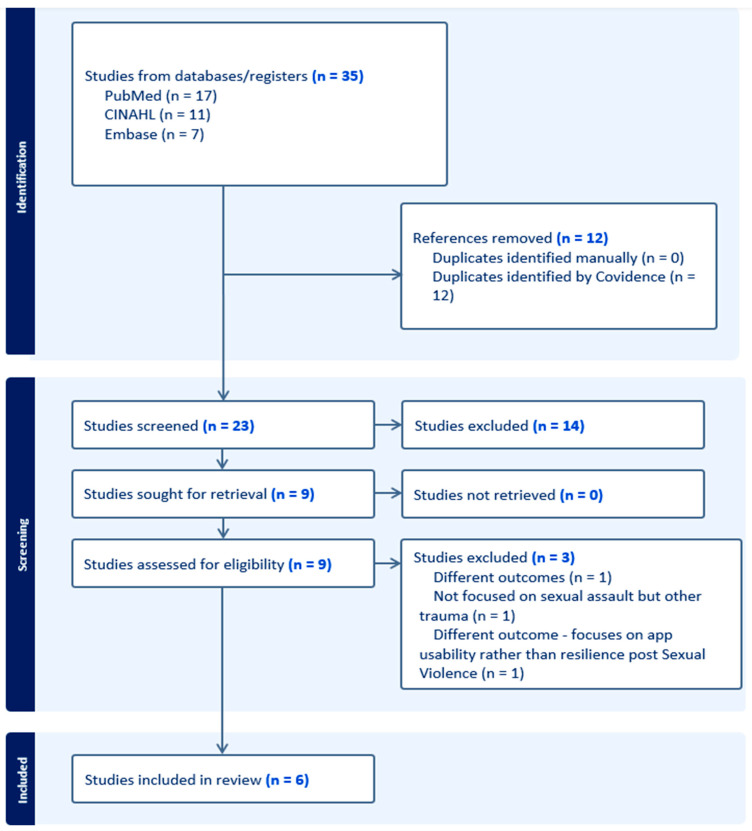
PRISMA Diagram Showing Article Screening Process.

Articles were uploaded into Covidence, a systematic review software for screening, review, and full-text evaluation [13]. Two reviewers independently screened titles, abstracts, and full texts using predefined inclusion and exclusion criteria (Table 1). Only primary research articles using a mixed-methods design and published in English between 2014 and 2024 were included. Mixed-methods reviews, methodology-focused articles, and papers with no full-text access were excluded. The full selection process is illustrated in the PRISMA diagram (Figure 1).

### 2.3. Quality Appraisal

Quality appraisal was conducted independently by two reviewers using a modified version of the Johns Hopkins Nursing Evidence-Based Practice (JHNEBP) tool. This tool includes 13 criteria used to rate the quality of MMR studies as high (A: scores 11–13), good (B: scores 7–10), or low (C: scores < 7). Only studies rated as A or B were included in the final synthesis.

### 2.4. Data Extraction and Analysis

A customized data extraction sheet was developed to capture study characteristics and review-specific variables including authors/year, study aims, rationale for using mixed methods, type and timing of integration, quantitative and qualitative data collection strategies, measurement tools used, and outcomes assessed (Table 2 and Table 3).

Two reviewers independently extracted data, and discrepancies were resolved through discussion. A content analysis approach, informed by the constant comparison method described by [11], was used to categorize and summarize patterns across studies. This analysis focused on recurring methodological choices, integration strategies, and implications for future trauma research.

## 3. Results

### 3.1. Study Characteristics

Only six studies met the inclusion criteria for this review, highlighting the limited application of mixed-methods approaches in resilience research following sexual assault. The six studies included in this integrative review were published between 2018 and 2022 and represented diverse participant populations, including survivors of sexual violence from varied age groups and cultural backgrounds. Across studies, participants included women and sexual minorities with histories of sexual assault, childhood sexual abuse (CSA), dating violence, or clergy abuse. Most studies focused on young adults and emerging adults, although one explored outcomes among older adult survivors. All studies applied both qualitative and quantitative approaches to understand aspects of resilience and healing after sexual assault.

Across the six studies included in this integrative review, several thematic areas emerged regarding the use of mixed methods to examine resilience after sexual assault. These included the qualitative and quantitative approaches employed, the rationales cited for adopting a mixed-methods design, the strategies used to integrate different forms of data, and the measures and outcomes assessed. The sections below describe findings across each of these thematic areas.

### 3.2. Theme: Qualitative and Quantitative Approaches

The qualitative strands across studies primarily involved semi-structured interviews, conducted either in person or online, which ranged from 45 to 90 min. In one study [17], qualitative data were collected via open-ended questions embedded within a survey, offering a less resource-intensive alternative. Most studies employed interviews to explore survivor narratives, coping strategies, and meaning-making around their trauma experiences.

Quantitative methods were primarily online surveys, reflecting the sensitive nature of the topic and the need for anonymity. These surveys measured a variety of constructs relevant to resilience, including PTSD, depression, anxiety, coping, and social support. Tools such as the CD-RISC2, PTSD Checklist (PCL), and ACE questionnaire were commonly used. Only two studies measured resilience directly using standardized scales; the others relied on proxy measures or developed context-specific instruments.

### 3.3. Theme: Rationales for Mixed-Methods Use

All six studies provided explicit rationales for choosing a mixed-methods approach. Three studies cited a completeness rationale [14,15,16], aiming to generate a fuller understanding by combining statistical prevalence with lived experience. Pooler & Barros-Lane used an enhancement rationale [17], explaining that qualitative data helped deepen the interpretation of quantitative findings. Saint Arnault & Sinko applied a confirm and discover rationale [18] by developing hypotheses from interviews and testing them quantitatively in the same sample. Finally, Timraz used a triangulation rationale to corroborate findings and enhance the credibility of the results [19].

Notably, the location of these rationales varied: some appeared in the Section 1, others in the Section 2 or Section 5. While all authors articulated a purpose for their mixed-methods design, few explicitly connected this rationale to their overall study structure.

### 3.4. Theme: Integration Strategies

Integration across the mixed-methods studies varied. Most commonly, authors used connecting strategies [14,15], linking data types through sampling or sequencing. Merging strategies, where quantitative and qualitative findings were analyzed together, were used by Pooler & Barros-Lane and Timraz [17,19]. One study applied a building approach, using qualitative data to inform survey design or interpretation [18]. Notably, joint display tables, a useful tool for visualizing integration, were used in only one study, suggesting an underutilization of visual synthesis methods. Below is a more detailed breakdown of how each study applied these strategies:

Pooler & Barros-Lane (2022) used a merging integration strategy, combining quantitative and qualitative data within a single online survey to explore resilience in women survivors of clergy-perpetrated sexual abuse [17]. The authors constructed a five-item resilience index based on a literature review, conversations with survivors, and clinical expertise and then quantitatively analyzed predictors of resilience. Qualitative integration occurred through an open-ended survey item asking participants what was most healing in their recovery. These responses were analyzed using a framework analysis to identify common recovery pathways. While analyzed separately, quantitative and qualitative results were brought together narratively in the discussion to underscore the multifaceted nature of healing and the importance of spiritual and clinical support systems.

Lachapelle et al. (2022) used a convergent, embedded mixed-methods design to explore cyber-dating violence (DV) among youth [16]. Integration occurred through a building approach: cyber-DV items were added to the survey only after this theme emerged in several interviews, allowing qualitative findings to inform the development of the quantitative instrument. The authors then analyzed survey data (n = 332) and thematically coded interviews (n = 16) independently, presenting both in parallel under a shared conceptual framework (the Resilience Portfolio Model), demonstrating a form of merging. The rationale for integration was completeness—using both datasets to provide a comprehensive picture of victimization risk factors and coping strengths.

Timraz (2018) employed a convergent mixed-methods design with the rationale of complementarity, aiming to gain a comprehensive understanding of coping and psychological outcomes among Arabic female CSA survivors [19]. Qualitative and quantitative data were collected concurrently from the same sample of 20 participants. Integration was achieved through merging, using a joint display matrix that presented both qualitative themes (e.g., coping strategies, cultural influences, disclosure reactions) and quantitative scores (e.g., depression, PTSD, acculturation) side by side for each participant. This joint display enabled cross-case comparisons to identify patterns of convergence or divergence between participants’ narratives and their survey outcomes. Although not analyzed statistically, the display facilitated narrative interpretation and thematic patterning across data types.

Saint Arnault and Sinko (2019) employed an exploratory sequential mixed-methods design with a rationale of development, aiming to generate and test theory about recovery processes among survivors of unwanted sexual experiences (USEs), with and without a history of childhood sexual assault (CSA) [18]. Integration was achieved through a building strategy: the authors conducted qualitative thematic analysis on narrative interviews (n = 24), abstracted initial codes into higher-order “complexes” (e.g., minimization, negative impact, hope and fulfillment), and used patterns in code frequencies to generate hypotheses. These hypotheses were then tested in a larger quantitative sample (N = 206) using standardized survey instruments. This approach reflects a structured and deliberate building process in which qualitative findings inform the subsequent quantitative analysis.

Ho et al. (2021) employed a mixed-methods design that combined both connecting and building integration strategies [15]. The connecting strategy was evident in the recruitment of qualitative interview participants as a nested subsample from the larger survey sample, enabling the researchers to link individual experiences with broader patterns in the quantitative data. Simultaneously, a building strategy was used, as a qualitative analysis generated thematic “complexes” that directly informed the development of hypotheses tested in the quantitative phase. This dual integration approach supported both a development rationale, by using qualitative findings to construct and operationalize key constructs (e.g., minimization, hope and fulfillment) for hypothesis testing, and a completeness rationale, by leveraging the strengths of both qualitative depth and quantitative breadth to offer a more comprehensive understanding of trauma recovery. The integration of findings occurred through a narrative comparison and regression analysis to examine relationships between constructs across both datasets.

Hequembourg et al. (2021) applied a mixed-methods approach to understand sexual victimization and coping across sexual identity groups [14]. The authors used a connecting integration strategy, where qualitative focus group findings informed the selection and adaptation of items for a subsequent quantitative survey, allowing earlier qualitative themes (e.g., identity-specific stressors and coping responses) to shape the quantitative phase. The rationale was completeness, aiming to gain a more holistic understanding of coping behaviors by combining the depth of qualitative insights with the breadth of survey data. While the study design linked phases sequentially, the integration remained mostly conceptual rather than procedural, with limited evidence showing how individual qualitative themes directly mapped onto specific survey constructs.

### 3.5. Theme: Measures and Outcomes

While most studies utilized commonly used instruments such as the PTSD Checklist, GAD-7, and ACE questionnaire, the psychometric validity of these tools was not always established for the specific survivor populations under study. For example, referene [15] employed the CD-RISC2, a resilience scale adapted for Chinese populations, yet its validation among sexual assault survivors remains limited. Similarly, referene [17] designed a context-specific measure for clergy abuse survivors. Although they reported acceptable internal consistency, the tool lacks a broader psychometric evaluation. These examples reflect broader challenges in ensuring methodological rigor when applying standardized tools to culturally and experientially distinct trauma populations.

Across the studies, additional outcomes such as depression, anxiety, social support, coping strategies, and trauma history were assessed using various instruments, including the Post-traumatic Growth Inventory (PTGI) and the Beck Depression Inventory (BDI-II). This diversity in measurement highlights both the multidimensional nature of resilience and the critical importance of aligning instruments with the context and characteristics of the population being studied.

In sum, while each study applied MMR to examine resilience after sexual assault, their specific research questions, rationale, integration methods, and measurement strategies varied widely. This variation underscores both the adaptability of mixed-methods approaches and the need for greater clarity and standardization in how these methods are justified and reported in trauma research.

## 4. Discussion

In this integrative review, six studies were identified that examined resilience and healing following sexual assault using mixed-methods approaches. Across these studies, semi-structured interviews and online surveys were the most common data collection methods, reflecting an effort to balance narrative depth with broad quantitative assessment. While all six studies provided rationales for using mixed methods—most commonly completeness and triangulation—their strategies for integrating data varied, and few employed joint displays or explicit mechanisms to link datasets meaningfully. Measures of resilience also differed widely, and population-specific validation of instruments was often lacking.

The central aim of this review was not to synthesize findings related to resilience per se, but to examine how mixed-methods research (MMR) was applied within the research context of resilience and healing following sexual assault. The limited number of eligible studies underscores the continued underutilization of MMR in this field. Given the complex and multidimensional nature of resilience—encompassing psychological, social, cultural, and structural components—mixed-methods approaches are particularly well suited to its investigation. As Creswell and Plano Clark emphasize, combining qualitative and quantitative methods enhances the richness of insights into complex phenomena, particularly when studying sensitive topics such as trauma [4]. Yet despite this potential, MMR remains a less common approach in sexual violence research, possibly due to training gaps, structural limitations in funding or publication outlets, or lingering preferences for single-method designs in the field.

Across the six studies, semi-structured interviews were the primary qualitative method, often conducted in person or online to explore survivors’ meaning-making, coping, and recovery processes. This aligns with the existing literature that highlights the appropriateness of semi-structured interviews in trauma research due to their flexibility and participant-centered nature [20,21]. One study collected qualitative data through open-ended survey responses [17], demonstrating the adaptability of MMR designs and the feasibility of less resource-intensive alternatives. Quantitative strands typically involved online surveys measuring constructs such as PTSD, depression, social support, and resilience, an approach consistent with best practices in trauma research that emphasize participant anonymity and accessibility [22].

All six studies articulated clear rationales for using mixed methods. Three studies used a completeness rationale [14,15,16], combining quantitative breadth with qualitative depth to provide a more holistic understanding of resilience. One study used an enhancement rationale [17], explaining that qualitative data enriched the interpretation of survey results. One study adopted a confirmation/discovery rationale, using qualitative data to generate hypotheses later tested quantitatively [18]. Finally, the last study used a triangulation rationale [19] to corroborate findings across data types. While all studies provided a rationale, few explicitly linked their rationale to the structure of their design or elaborated on how integration supported that rationale.

Integration strategies also varied significantly across the studies. Two studies used connecting strategies [14,15], where qualitative participants were drawn from the quantitative sample or where qualitative themes shaped subsequent survey content. Merging strategies where qualitative and quantitative data were analyzed together or side by side were used by two studies [17,19]. Notably, Timraz was the only study to use a joint display [19], organizing qualitative themes and quantitative scores for each participant in a matrix that allowed basic narrative interpretation. However, even in that case, integration remained more descriptive than analytical. Building strategies were used by two studies [15,18], who developed and tested hypotheses based on qualitative findings. In Ho et al., both building and connecting were used in tandem [15], suggesting a more deliberate integration process. Still, only a few studies provided detailed accounts of how integration occurred in practice, and most did not go beyond narrative triangulation in their final interpretations. This reflects a broader trend in MMR where integration is conceptually acknowledged but procedurally underdeveloped [23].

Measurement strategies also varied, and while most studies employed standardized tools such as the PTSD Checklist (PCL-5), the Generalized Anxiety Disorder scale (GAD-7), and the ACE questionnaire, concerns remain about the cultural and contextual validity of these instruments. For instance, Ho et al. used the CD-RISC2 [15], a resilience tool adapted for Chinese populations, yet its validation among sexual assault survivors was not clearly established. Pooler & Barros-Lane developed a context-specific measure tailored to clergy abuse survivors, but while they reported acceptable internal consistency, the tool has not undergone broader psychometric testing [17]. These examples highlight a critical challenge in MMR trauma research: balancing the need for generalizable measures with the importance of cultural and experiential specificity [24,25]. Ensuring that tools are not only psychometrically sound but also meaningful to the populations under study is essential for advancing trauma-informed care and understanding.

These findings have practical implications for both research and mental health nursing practice. For researchers, the review underscores the need for clearer articulation and execution of integration strategies, including the use of joint displays and transparent descriptions of how data strands relate to one another. It also highlights the need for greater attention to instrument validation, particularly when working with culturally distinct or trauma-affected populations. For mental health nursing and allied disciplines, integrating survivors’ narratives with measurable constructs offers a richer, more grounded understanding of resilience pathways. This can inform clinical assessments, guide the design of trauma-informed interventions, and support the development of culturally responsive care models that reflect the lived experiences of diverse survivor groups.

In sum, this review demonstrates that while MMR holds significant promise for studying resilience following sexual assault, its application remains uneven. To fully realize the benefits of methodological pluralism in trauma research, future studies should move beyond merely asserting integration and strive for deeper, more systematic connections between data strands. Doing so will strengthen the rigor, relevance, and impact of resilience research in this critically important field.

## 5. Conclusions and Recommendations

MMR offers a valuable but underutilized approach to studying resilience after sexual assault. This review highlights that, while MMR is being applied intentionally in this field, its use remains relatively limited. The studies included in this review used mixed methods not simply as a preference but as a deliberate strategy to capture the complex and layered experiences of survivors. Despite variability in design and measurement, a common goal was the integration of different types of data to reflect both breadth and depth. However, more consistent use of structured integration strategies, particularly visual tools like joint displays, could improve the transparency, interpretability, and practical impact of future research.

For mental health nursing, these findings underscore the need for research that reflects the real-world complexity of trauma recovery. Nurses often support survivors in both clinical and community settings, yet many assessment tools fall short of capturing survivors’ full emotional and relational healing journeys. Mixed-methods approaches offer an opportunity to close this gap by combining measurable indicators with survivors’ voices and lived realities.

To strengthen the quality and consistency of future MMR in this area, we recommend the following:

- Clarify Rationale and Align with Research Questions: Authors should explicitly state why a mixed-methods approach is needed and ensure that the rationale (e.g., completeness, triangulation) is clearly aligned with the research aims. This enhances methodological transparency and helps readers evaluate the study’s coherence.
- Use Qualitative Data Strategically in Nursing Contexts: In trauma-informed nursing research, qualitative strands should go beyond thematic exploration. Narrative data can directly inform clinical frameworks, communication strategies, and culturally responsive care. Nurses need access to these insights to guide patient-centered practice.
- Select Quantitative Measures Thoughtfully: Researchers should prioritize validated, trauma-relevant, and culturally appropriate instruments for measuring resilience and associated outcomes. When standardized tools are not adequate, the development of context-specific measures should be clearly justified and described.

Future studies should also broaden the geographic and cultural scope of MMR in this field and adopt clearer integration methods that maximize insight across data types. With stronger rationale, thoughtful measure selection, and more intentional integration, MMR can play a central role in advancing trauma-informed care for diverse survivor populations.

## 6. Limitations

This review included only six studies, which may not fully capture the diversity of mixed-methods research (MMR) approaches used to study trauma recovery and resilience. Additionally, most of the included studies were conducted in North America, which limits the global applicability and cultural generalizability of the findings. Another limitation is the restriction to English-language publications, which may have excluded relevant studies conducted in other languages and cultural contexts. Furthermore, only four databases (PubMed, CINAHL, PsycINFO, and EMBASE) were searched, which may have limited the comprehensiveness of the review and contributed to the small number of eligible studies.

## Figures and Tables

**Table 1:** Inclusion and exclusion criteria.

	Inclusion	Exclusion
1.	Published from 2014 to 2024	No full article available
2.	Published in English	Published in methodology journals
3.	Mixed-methods primary research	Methodology focused articles
4.	“A” and “B” quality papers after quality appraisal *	Mixed-methods reviews

* A = high quality (score 11–13); B = good quality (score 7–10) based on the modified JHNEBP tool.

**Table 2:** Data extraction—qualitative and quantitative strands.

Author/ Year	Qualitative Strand		Quantitative Strand		
	Article Participants Characteristics	Qualitative Aim/Method Used	Quantitative Aim Method of Data Collection	Quantitative Scales Used	Outcomes Measured
Hequembourg et al. (2021) [14]	Canada; N = 55; women aged 18–60+; diverse trauma types including child sexual abuse, IPV, and adult sexual assault; various service use histories.	Semi-structured interviews	To assess the prevalence and severity of women’s lifetime SV Survey	Child Sexual Abuse (CSA) Adult Sexual Victimization	Sexual victimization prevalence Coping strategies Disclosure experiences
Ho et al. (2021) [15]	Hong Kong; N = 433 (quantitative) + 34 (qualitative); ages 18–24; university students; gender not fully specified, but both male and female included; trauma type: Adverse Childhood Experiences (ACEs) including abuse, neglect, and household dysfunction.	To assess the contribution of resilience in explaining relationships between impaired mental health and ACEs Semi-structured interviews	To examine the associations between negative mental health outcomes and ACEs among young adults Online survey	WHO ACE-international questionnaire CD-RISC2 Hospital Anxiety and Depression Scale (HADS) Adjustment Disorder New Module (ADNM-20) International Classification of Diseases (ICD-11)	Adverse childhood experiences Resilience Anxiety and depression Maladjustment and current life stressors Post-traumatic stress
Lachapelle et al. (2022) [16]	Canada; N = 332 (quantitative), 16 (qualitative); age = 14–25 years (quantitative M = 19.90, SD = 3.23; qualitative M = 16.56, SD = 0.81); gender = predominantly cisgender females (74.7%); trauma type = childhood maltreatment including neglect, emotional abuse, sexual abuse, and exposure to interparental violence; population = adolescents and emerging adults, primarily students.	To bring a positive overview in terms of the strengths contained by victimized youth and how they translate into their personal and relational lives Semi-structured interviews, face to face or online	To provide insight into the specific vulnerability mechanisms involved in cyber-DV compared to other types of DV. Online survey	Cyber Aggression in Relationships scale (CARS) Revised Conflict Tactics Scale Early Trauma Inventory Self Report Quebec Child and Adolescent Health and Social Survey (ESSEA)	Cyber-DV victimization Child maltreatment Perceived social support
Pooler & Barros-Lane (2022) [17]	United States; N = 159 (mixed methods); mean age at time of study: 47 years (SD = 12); mean age at time of abuse: 33 years (SD = 10); majority cisgender women, primarily Caucasian (81%), with smaller representation from African American (11.3%), Hispanic (3.1%), Asian (0.6%), Pacific Islander (0.6%), and other (2.5%); trauma type: clergy-perpetrated sexual abuse beginning at age 16 or older.	What factors are most helpful in healing? Open-ended question in online survey	What factors most contribute to resilience after trauma? Online survey	PTSD symptom checklist Measures created for the sample (non-standardized measure)	PTSD Experiences of abuse Resilience
Saint Arnault & Sinko (2019) [18]	United States; N = 273 (quantitative) + 24 (qualitative); university-based sample of women aged 18+ who experienced unwanted sexual encounters as undergraduates or within 5 years post-graduation; gender: All participants self-identified as female; trauma type: unwanted sexual encounters (USE) during university, with subgroup comparisons between survivors with and without childhood sexual abuse (CSA) histories.	To identify how contextual and internal processing impacted healing after unwanted sexual experiences, as well as the nature and meaning of healing for the survivors. Narrative interview	Hypotheses Survivors of both USE and CSA will have less minimization than survivors of USE alone. Survivors of both USE and CSA will have higher negative impacts than survivors of USE alone. Survivors of both USE and CSA will have more hope and fulfilment. Online survey	PTSD checklist for DMV (PCL-5) Patient Health Questionnaire (PHQ-8) Generalized Anxiety Disorder (GAD-7) Adverse Childhood Experiences (ACE) Centrality of Events Scale–short form Post-traumatic Growth Inventory (PTGI) Sense of Coherence (SOC) Self-compassion Scale (SCS)	PTSD Depression Anxiety Childhood sexual assault Minimization Hope and fulfilment
Timraz (2018) [19]	United States; N = 20; Arabic female survivors of childhood sexual abuse (CSA); age: 18+ (specific age range not reported); gender: All participants identified as female; trauma type: CSA prior to age 17; sample recruited through a university webpage, social media, flyers, and word of mouth.	To explore the CSA characteristics, perception, and coping strategies of female survivors of Arabic descent Semi-structured interviews, face to face, email, phone interview	Explore the long-term psychological outcomes of CSA and the survivors’ perception of how their coping strategies facilitate or limit their psychological adjustment during adulthood. Questionnaire answered at the same time as interviews	Acculturation rating scale for Arab Americans (ARSAA-IIE) Social reactions questionnaire (SRQ/SRQ-CSA) Ways of coping questionnaire (WCQ-R) Posttraumatic diagnostic scale (PDS-5) Beck Depression Inventory (BDI-II)	Acculturation Social reaction to abuse disclosure. Coping PTSD Depression

**Table 3:** Data extraction table—rationale and integration.

Author/Year	Mixed-Methods Aims	Rationale (Bryman, 2006) [10]			Integration (Fetters et al., 2013) [9]	
		Article Rationale Statement	Typology	Where Cited in Paper	Type of Integration	Joint Display Table Used
Hequembourg et al. (2021) [14]	To utilize a mixed-methods approach to understand SMW’s recovery process	“The present study used quantitative—and when applicable—qualitative methods to understand lesbian, bisexual, and heterosexual women’s SV experiences.” p. 4	Completeness	Methods section	Connecting	No
Ho et al. (2021) [15]	To explore culture specific factors that may explain or influence resilience in the context of ACEs.	“We utilized a mixed methods approach to generate a fuller understanding of ACEs, resilience, and mental health outcomes from a sample of young Chinese adults” p. 4	Completeness/development	Introduction	Connecting/building	No
Lachapelle et al. (2022) [16]	To examine the association between risk and protective factors and the risks of cyber-DV victimization in adolescence and emerging adulthood.	“The mixed methods design provides a deeper understanding of the abilities, strengths, or support-seeking process mobilized by victimized youth to adapt to cyber DV”. p. 603	Completeness	Strengths and limitations	Building/merging	No
Pooler & Barros-Lane (2022) [17]	To shed more light on women who had survived clergy sexual abuse and their experiences, and to inform social work practice by exploring what these women found most helpful in healing.	“We wanted to not only know who they were, but also to learn more about their experiences of being abused, how the church responded, and how they healed.” p. 126	Enhancement	Literature review/ background	Merging	No
Saint Arnault & Sinko (2019) [18]	This study aims to compare healing experiences for a sample of sexual assault survivors who did, or did not, also experience childhood sexual assault.	“Using mixed methods enhances our understanding of these relationships among the interview respondents by testing hypotheses in the same study.” p. 2. “We believe that this approach has value because it allows us to listen deeply to the experiences and perspectives of assault survivors and then use this data to explore trends in a larger dataset in the same study.” p. 8	Confirm and Discover	Introduction Future directions	Building	No
Timraz (2018) [19]	This mixed-methods study addresses Arabic women’s coping with their experiences of CSA and long-term psychological outcomes.	“The aim for collecting both qualitative and quantitative data is to corroborate the results of the two types of data and to bring greater insight into the problem that would not be obtained separately from either type of data.” p. 6	Triangulation	Purpose of the study	Merging	Yes

## Data Availability

The data used in this integrative review were derived from previously published studies, which are cited throughout the manuscript. No new data were generated or analyzed in this study.

## Public Involvement Statement

No public involvement in any aspect of this research.

## Guidelines and Standards Statement

This manuscript was drafted against the Whittemore and Knafl integrative review methodology [11].

## Abbreviations

The following abbreviations are used in this manuscript:

MMR	Mixed-Methods Research
WHO	World Health Organization
